# A randomized trial of once daily versus twice daily dosing of oral iron in CKD

**DOI:** 10.1038/s41598-022-26589-x

**Published:** 2023-01-04

**Authors:** Vivek Sood, Kajal Kamboj, Prateek Bhatia, Vishal Sharma, Monica Kundu, Arpita Ghosh, Sanjay Kumar Singh, Thakur Sen, Prabhjot Kaur, Raja Ramachandran, Manish Rathi, Harbir Singh Kohli, Krishan Lal Gupta, Samir Malhotra, Ashok Kumar Yadav, Vivek Kumar, Vivekanand Jha

**Affiliations:** 1 Department of Nephrology, Postgraduate Institute of Medical Education and Research, Chandigarh, 160012 India; 2 Department of Paediatrics, Postgraduate Institute of Medical Education and Research, Chandigarh, India; 3 Department of Gastroenterology, Postgraduate Institute of Medical Education and Research, Chandigarh, India; 4 George Institute for Global Health, New Delhi, India; 5 Department of Pharmacology, Postgraduate Institute of Medical Education and Research, Chandigarh, India; 6 Department of Experimental Medicine and Biotechnology, Postgraduate Institute of Medical Education and Research, Chandigarh, India; 7 School of Public Health, Imperial College, London, UK; 8 Manipal Academy of Higher Education, Manipal, India

**Keywords:** Nephrology, Kidney diseases

## Abstract

We investigated the effect of two dosing regimens of oral iron on iron status and hematological parameters in patients with CKD. In this single center, open label, randomized, active controlled clinical trial, stable adult patients with CKD stage G3–4 with percentage transferrin saturation (%TSAT) ≤ 30% and serum ferritin ≤ 500 ng/ml were eligible. Participants were randomized to receive either 100 mg of ferrous ascorbate once daily (OD group) or 100 mg of ferrous ascorbate twice daily (BD group, total daily dose 200 mg). The primary outcome was change in %TSAT between groups over 12 weeks. The secondary outcomes were changes in other iron status and hematological parameters, serum interleukin-6 (IL-6) and hepcidin. 80 participants were enrolled out of which 76 completed the study. Change in %TSAT was not significantly different between groups (β = − 1.43, 95% CI − 3.99 to 1.12, BD group as reference). The rise in serum ferritin was less in the OD group as compared to BD group (β = − 0.36, 95% CI − 0.61 to − 0.10) whereas MCHC increased in the OD group as compared to decrease in the BD group (β = 0.37, 95% CI 0.067–0.67). These observations need exploration to ascertain the impact of different oral iron dosing strategies in CKD.

## Introduction

Anemia is common in chronic kidney disease (CKD) and has been associated with impaired quality of life, cardiovascular disease (CVD) and mortality^1,2^. Iron deficiency is common in patients with CKD and an important modifiable factor in treatment of anemia^3^. The current guidelines recommend trial of iron supplementation in CKD when increase in hemoglobin concentration or decrease in erythropoietin dose is desired and investigations reveal percentage transferrin saturation (%TSAT) ≤ 30% and serum ferritin ≤ 500 ng/ml^4,5^. The absorption of iron from gastro-intestinal tract and release of iron from reticuloendothelial cells for erythropoiesis are tightly regulated through hepcidin–ferroportin axis^6^. Hepcidin concentrations increase in response to iron excess and inflammation. CKD is associated with elevated hepcidin and ferritin concentrations due to underlying inflammation, independent of iron status^7^. Ascertaining a state of functional iron deficiency that reflects impaired utilization of iron for erythropoiesis despite apparent stores and predicting erythropoietic response to iron supplementation in patients with CKD are clinical challenges. Other parameters like percentage of hypochromic red blood cells, reticulocyte hemoglobin content and soluble transferrin receptor have been suggested as markers of true iron deficiency in CKD but are either not widely available or have not been found useful^5,8^.

In patients with CKD who are not on dialysis, oral route may be preferred as initial mode of iron supplementation. Oral iron is inexpensive, self-administered, convenient and easily available. The dosing frequency varies from once a day to 2–4 times a day depending on type of preparation^5^. Less frequent dosing might lead to better compliance, decreased costs and less drug related gastro-intestinal side effects. Recently, it has been shown that oral administration of iron in the morning increases circulating hepcidin concentrations and thus, impairs absorption of iron from subsequent doses in the day^9^. This has led to discussion about using alternate day oral dosing. The superimposed effect of oral iron supplementation on already elevated hepcidin (as in patients with CKD) and subsequently, on oral iron absorption is not known. We investigated the effect of once or twice a day dosing of oral iron formulation on measures of iron sufficiency in patients with stage 3–4 CKD through a randomized controlled trial.

## Methods

### Study design

The study was a single centre, pilot study. It was parallel arm, randomized, open label, active controlled, interventional trial conducted at the Postgraduate Institute of Medical Education and Research (PGIMER), Chandigarh, India. The trial was approved by the Institute Ethics Committee of PGIMER, Chandigarh and prospectively registered at the Clinical Trials Registry of India (www.ctri.nic.in, trial registration number CTRI/2017/02/007799, date of registration 07/02/2017). This study was conducted in accordance with Declaration of Helsinki.

### Study population

All adults with a diagnosis of CKD stage G3–4 attending the outpatient clinic at PGIMER were eligible for screening. Subjects between the ages of 18 and 70 years, TSAT ≤ 30%, serum ferritin ≤ 500 mg/L^4^ and clinically stable course for the last 3 months as judged by treating physician were included. Exclusion criteria were blood hemoglobin < 10 g/dl, current use or use within last 3 months of oral or intravenous iron preparations, suspected or known diagnosis of autoimmune disease, past or present diagnosis of malignancy, known diagnosis of chronic liver disease, bleeding diathesis, history of gastro-intestinal surgery, present or past diagnosis of peptic ulcer disease, gastro-intestinal bleeding or any form of gastro-intestinal disease predisposing to malabsorption, history of allergic reactions to any form of oral or intravenous iron preparations, current intake of gastric acid inhibitors (histamine receptor blockers, proton pump inhibitors), history of prior non-compliance with oral iron therapy due to gastro-intestinal side effects, current use or use within last 6 months of immunosuppressive drug therapy, pregnant or lactating females and life expectancy < 1 year. All patients provided written informed consent before enrolment.

### Enrolment and randomization

At enrolment, all participants entered 2-week run-in period. This was done to ensure clinically stable status of participants. The visit scheduled after completion of run-in period was referred to as baseline visit. Participants were randomized in 1:1 allocation ratio to either of the two study groups at baseline visit. The randomization scheme was generated from the website www.randomization.com with identifying seed number 12459. Allocation concealment was done by sequentially numbered, sealed, opaque envelopes.

### Intervention and follow up

Participants were randomized to oral iron supplementation with ferrous ascorbate in either once daily (100 mg of elemental iron once daily i.e., 100 mg total daily dose, referred to as OD group) or twice daily (100 mg elemental iron twice daily i.e., 200 mg total daily dose, referred to as BD group) dosing for 12 weeks. They were instructed to take drug empty stomach at least 1 h prior to the meals. Participants were scheduled for follow up visits at 2, 5 and 12 weeks after baseline visit. Participants were deemed compliant if they had taken > 90% of the dispensed doses. Participants were encouraged to report any change in their health status till 2 weeks after completion of study period. All adverse events were recorded. Fasting blood samples were collected at baseline, 2 weeks, 5 weeks and 12 weeks. Samples collected at baseline and 12 weeks were also processed for storage at − 80 °C.

### Outcomes

The primary outcome was difference in change in % TSAT over 12 weeks between the two groups. The secondary outcomes were differences in changes in serum ferritin, blood hemoglobin concentrations, mean corpuscular volume (MCV), mean corpuscular hemoglobin (MCH), mean corpuscular hemoglobin concentration (MCHC), percentage of hypochromic red blood cells (% HYPO-He), reticulocyte hemoglobin equivalent (RET-He), serum interleukin-6 (IL-6) and serum hepcidin concentrations over 12 weeks between the groups.

### Measurements

All measurements were done in fasting blood samples. Blood hemoglobin concentrations, MCH, MCV, MCHC, % HYPO-He and RET-He were measured at every visit on Sysmex XN-1000™ Hematology Analyzer (Sysmex Corporation, Kobe, Japan). These measurements were done within 4 h of sample collection. % TSAT concentrations were calculated from serum iron^10^ and total iron binding capacity (TIBC)^11^ values measured manually based on standardized International Council for Standardization in Haematology (ICSH) methods. Serum ferritin concentrations were measured at every visit on ADVIA Centaur^®^ CP Immunoassay System (Siemens Healthcare, Erlangen, Germany) by chemiluminescence. As part of quality assurance in laboratory practices, routine controls (high, low, normal) are run daily as per manufacturer recommendations. Standard internal quality control and calibration practices are followed for all analytes. Serum IL-6 (Quantikine^®^ solid phase sandwich ELISA; R&D Systems, Minneapolis, MN, USA) and serum hepcidin (Quantikine^®^ solid phase sandwich ELISA; R&D Systems, Minneapolis, MN, USA) were measured in stored serum samples collected at baseline and 12 weeks.

### Statistical considerations

Assuming dropout rate of 10%, a sample size of 80 participants (40 in either group) was required to detect absolute difference of 3% in % TSAT [standard deviation (SD) ± 4.5%] between the two groups with 80% power and two sided α of 0.05. All participants with non-missing outcome data were included in the analysis. Descriptive statistics were used to describe characteristics of study participants. Data were presented as mean ± SD or median (interquartile range, IQR) as appropriate. For baseline measurements, continuous variables were compared by independent sample t test if normally distributed, or Mann–Whitney U test if distribution were skewed. Categorical variables were analyzed by Chi-squared test or Fisher exact test as appropriate. We used linear mixed effect model as primary analysis to assess the difference in change in concentrations of primary and secondary outcomes over the study period between the 2 groups. Group (OD and BD), follow up time (week), sex and baseline concentration (value) were defined as fixed effects and study participants as random effects. Simple linear regression was modelled for those parameters which were measured at baseline and 12 weeks (IL-6 and hepcidin). P-values < 0.05 were considered statistically significant. Logarithmic transformation was used before fitting linear mixed effects model or simple linear regression model for following parameters: serum ferritin, %HYPO-He, TIBC, serum IL-6 and serum hepcidin.

### Ethics approval and consent to participate

Study was approved from the Institute Ethics Committee and patients were enrolled in this study after getting the written consent prior to study procedures.

## Results

**Figure 1:**
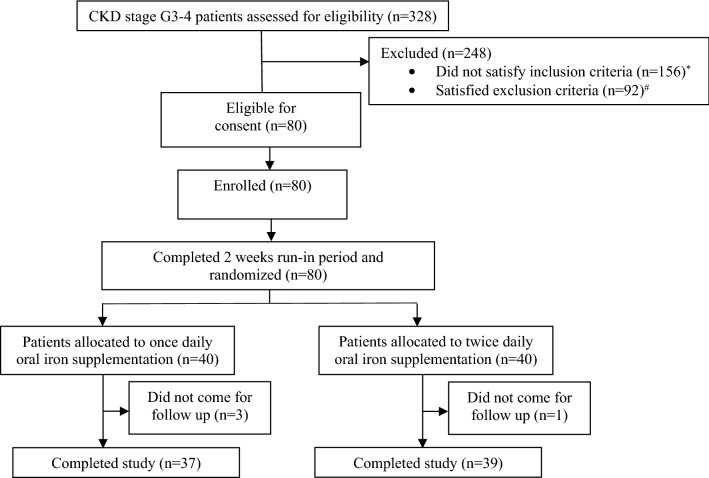
Study enrolment and follow up. *Subjects who did not satisfy inclusion criteria (n = 156): age 18–70 years (n = 6), eGFR by CKD-EPI 15–60 ml/min/1.73 m^2^ (n = 84), serum TSAT concentrations ≤ 30% and serum ferritin ≤ 500 mg/l (n = 49), clinically stable course as judged by the treating physician for last 3 months (n = 17). ^#^Subjects who satisfied exclusion criteria (n = 92): blood hemoglobin < 10 g/dl (n = 36), current use or use within last 3 months of oral or intravenous iron preparations (n = 17), suspected or known diagnosis of autoimmune disease (n = 6), past or present diagnosis of malignancy (n = 2), known diagnosis of chronic liver disease (n = 4), gastro-intestinal bleeding or any form of gastro-intestinal disease predisposing to malabsorption (n = 3), history of allergic reactions to any form of oral or intravenous iron preparations (n = 1), current intake of gastric acid inhibitors (H2 receptor blockers, proton pump inhibitors) (n = 16), history of prior non-compliance with oral iron therapy due to gastro-intestinal side effects (n = 5), current use or use within last 6 months of immunosuppressive drug therapy (n = 2).

A total of 328 patients with CKD stage G3-4 were screened (Fig. 1), and 80 were enrolled and randomized. A total of 37 participants in the OD group and 39 participants in the BD group completed the study protocol. Enrolment started in February 2017 and the last study follow up was completed in February 2019. Baseline characteristics and investigations (Tables 1, 2) were similar in both groups except for hemoglobin (12.59 ± 1.67 vs.11.91 ± 1.25 g/dl in OD vs. BD, respectively; p = 0.04) and estimated glomerular filtration rate (eGFR) by creatinine based chronic kidney disease epidemiology collaboration equation (CKD-EPI_Cr_) (39.51 ± 12.35 vs. 33.80 ± 11.10 ml/min/1.73 m^2^ in OD vs. BD, respectively; p = 0.03). The groups were similar with respect to sex, duration of CKD, use of angiotensin converting enzyme inhibitors/angiotensin receptor blockers or phosphate binders.

**Table 1:** Baseline characteristics of the study participants.

Parameters	OD (n = 40)	BD (n = 40)
**Sex (%)**		
Male	32 (80%)	24 (60%)
Female	08 (20%)	16 (40%)
Age (years)	51.5 ± 8.6	48.4 ± 13.2
BMI (kg/ m^2^)	25.5 ± 4.3	24.9 ± 4.7
**Diet (%)**		
Vegetarian	26 (65%)	29 (72.5%)
Mixed	14 (35%)	11 (27.5%)
**Stage of CKD (%)**		
Stage 3	29 (72.5%)	25 (62.5%)
Stage 4	11 (27.5%)	15 (37.5%)
**Cause of CKD (%)**		
Diabetic Kidney Disease	10 (25%)	6 (15%)
Chronic Glomerulonephritis	6 (15%)	2 (5%)
Chronic Interstitial Nephritis	9 (22.5%)	10 (25%)
Others	15 (37.5%)	22 (55%)
Time since diagnosis of kidney disease (months)	35.1 ± 38.3	23.5 ± 27.5
**Co-morbidities**		
Hypertension	35 (87.5%)	35 (87.5%)
Diabetes mellitus	11 (27.5%)	10 (25%)
Coronary artery disease	03 (7.5%)	02 (5%)
Renal stone disease	06 (15%)	04 (10%)
Primary Hypothyroidism	05 (12.5%)	04 (10%)
Others	04 (10%)	03 (7.5%)
**Medications**		
ACEi/ARBs	26 (65%)	19 (47.5%)
Calcium channel blockers	16 (40%)	17 (42.5%)
Beta blockers	10 (25%)	10 (25%)
Alpha blockers	07 (17.5%)	05 (12.5%)
Diuretics	12 (30%)	15 (37.5%)
Calcium based phosphate binders	04 (10%)	05 (12.5%)
Non-calcium based phosphate binders	02 (5%)	04 (10%)
Cholecalciferol supplementation	12 (30%)	20 (50%)
Uric acid lowering drug	09 (22.5%)	09 (22.5%)
Anti-platelet drugs	11 (27.5%)	10 (25%)
Statins	18 (45%)	20 (50%)
Sodium bicarbonate	26 (65%)	32 (80%)

Data presented as number (%) or mean ± standard deviation as appropriate.

*ACEi* angiotensin converting enzyme inhibitors, *ARBs* angiotensin II receptor blockers, *BMI* body mass index, *CKD* chronic kidney disease, *eGFR* estimated glomerular filtration rate.

**Table 2:** Baseline hematological and biochemical parameters in the study participants.

Parameters	OD (n = 40)	BD (n = 40)	p-value
% TSAT	20.5 (14.0, 25.0)	19.5 (15.3, 24.6)	0.90
Serum ferritin (µg/l)	77.9 (36.7, 153.6)	67.5 (46.6, 118.3)	0.84
Serum iron (µg/dl)	71.3 ± 28.4	69.4 ± 21.7	0.75
TIBC (µg/dl)	376.5 (297.5, 384.0)	380.0 (336.8, 386.0)	0.46
Hemoglobin (g/dl)	12.6 ± 1.7	11.9 ± 1.3	0.04
MCV (fL)	87.3 (83.2, 90.2)	86.7 (82.8, 89.7)	0.87
MCH (pg)	27.6 (25.7, 28.7)	27.3 (26.7, 28.9)	0.79
MCHC (g/dl)	31.4 ± 1.2	31.8 ± 1.2	0.17
% HYPO-He	0.3 (0.2, 0.9)	0.4 (0.2, 0.9)	0.60
RET-He (pg)	30.7 (29.1, 31.8)	30.6 (29.2, 31.7)	0.99
Serum IL-6 (pg/ml)	3.9 (1.3, 7.5)	3.3 (1.9, 11.8)	0.89
Serum hepcidin (ng/ml)	16.5 (8.4, 23.3)	18.5 (9.0, 25.3)	0.87

Data presented as mean ± standard deviation or median (25th, 75th percentile) as appropriate. Compared using the Mann–Whitney U test.

*% HYPO-He* percentage of hypochromic red blood cells, *IL-6* interleukin-6, *MCH* mean corpuscular hemoglobin, *MCHC* mean corpuscular hemoglobin concentration, *MCV* mean corpuscular volume, *RET-He* reticulocyte hemoglobin equivalent, *TIBC* total iron binding capacity, *% TSAT* percentage transferrin saturation.

### Change in iron status and hematological parameters in the study population

Serial measurements done at 2, 5 and 12 weeks in both the groups are shown in Supplementary Tables 1 and 2. Overall, %TSAT, serum ferritin, serum iron, MCV, MCH and serum hepcidin concentrations increased while TIBC decreased in the study population over 12 weeks (Supplementary Table 3). Serum IL-6 concentrations did not significantly change over 12 weeks in the overall study population.

### Changes in primary and secondary outcome parameters between groups

**Figure 2:**
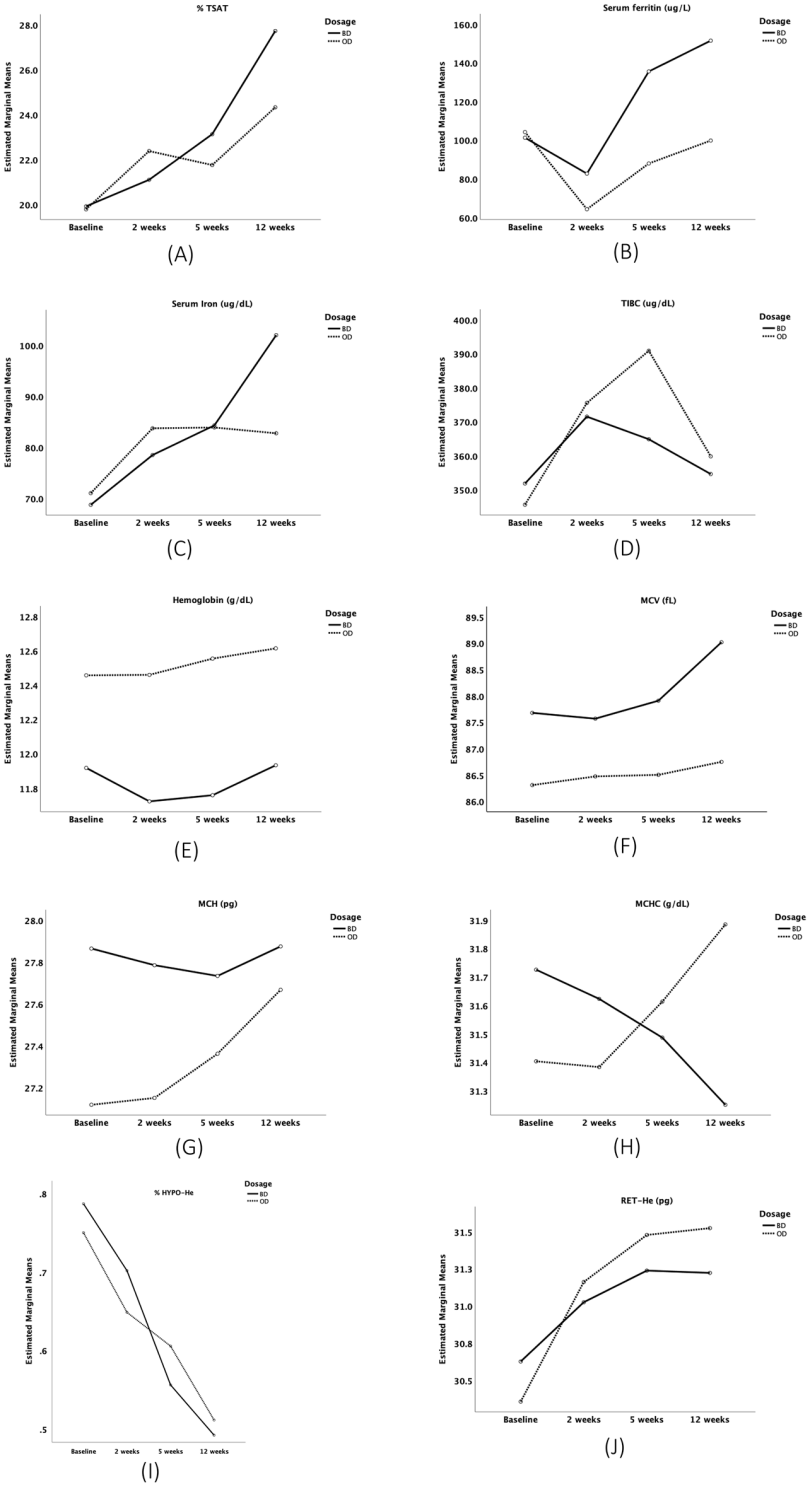
Change in estimated marginal means in the study groups. Trends for estimated marginal means for (**A**) %TSAT, (**B**) serum ferritin, (**C**) serum iron, (**D**) TIBC, (**E**) hemoglobin, (**F**) MCV (**G**) MCH, (**H**) MCHC, (**I**) % HYPO-He, (**J**) RET-He over 12 weeks. *% HYPO-He* percentage of hypochromic red blood cells, *MCH* mean corpuscular hemoglobin, *MCHC* mean corpuscular hemoglobin concentration, *MCV* mean corpuscular volume, *RET-He* reticulocyte hemoglobin equivalent, *TIBC* total iron binding capacity, *% TSAT* percentage transferrin saturation.

There was no significant difference in change in %TSAT at 12 weeks between the groups (Table 3, Fig. 2). Increase in serum ferritin concentration was significantly higher in the BD group as compared to OD group (Table 3, Fig. 2). The change in MCHC was significantly higher in the OD group as compared to the BD group (Table 3, Fig. 2). Serum iron, TIBC, hemoglobin, MCV, MCH, %HYPO-He, RET-He, serum IL-6 and serum hepcidin did not show any significant difference between the groups over 12-week study period (Table 3).

**Table 3:** Parameter estimates of the linear mixed effect model for change in primary and secondary parameters between OD versus BD group.

Parameters	Coefficients	Confidence interval	p-value
% TSAT	− 1.4	− 3.99, 1.12	0.27
Serum ferritin (log µg/l)^a^	− 0.36	− 0.61, − 0.10	0.01
Serum iron (µg/dl)	− 6.16	− 15.60, 3.28	0.20
TIBC (log µg/dl)^a^	0.04	− 0.04, 0.12	0.32
Hemoglobin (g/dl)	0.32	− 0.05, 0.69	0.09
MCV (fL)	− 0.34	− 1.40, 0.72	0.53
MCH (pg)	0.18	− 0.27, 0.63	0.43
MCHC (g/dl)	0.37	0.067, 0.67	0.02
% HYPO-He^a^	0.09	− 0.14, 0.32	0.45
RET-He (pg)	0.39	− 0.36, 1.15	0.31
Serum IL-6 (log pg/ml)^a,b^	− 0.18	− 0.93, 0.57	0.63
Serum hepcidin (log ng/ml)^a,b^	− 0.06	− 0.31, 0.19	0.63

Compared using the linear mixed effect model, BD group as reference.

*% HYPO-He* percentage of hypochromic red blood cells, *IL-6* interleukin-6, *MCH* mean corpuscular hemoglobin, *MCHC* mean corpuscular hemoglobin concentration, *MCV* mean corpuscular volume, *RET-He* reticulocyte hemoglobin equivalent, *TIBC* total iron binding capacity, *% TSAT* percentage transferrin saturation.

^a^Logarithmic transformation has been used for these parameters.

^b^Parameter estimates of simple linear regression model.

### Adverse events and others

All participants who completed study were compliant with study medication. Minor adverse events were recorded during the study (Supplementary Table 4). There were no events necessitating discontinuation of the drug or hospitalization.

## Discussion

In our study, the changes in %TSAT, serum iron and TIBC did not differ significantly between OD and BD dosing groups over 12 weeks. However, serum ferritin increased significantly in the BD group as compared to the OD group. Either repletion of intracellular stores or sequestration of iron within the cellular stores will lead to rise in serum ferritin concentrations. Overall, in the study population, serum hepcidin concentrations increased significantly over 12 weeks though there was no difference between the groups. This is in line with expected biological response in hepcidin concentrations to oral iron supplementation and repletion^6^. In normal individuals, increase in circulating hepcidin concentrations in response to oral iron load lead to impaired absorption from subsequent doses during the same day or next day^9,12^. But the hepcidin regulation is altered in CKD—concentrations are already elevated and whether or not they respond in the same way to oral iron is not known. We have neither done repeated serum hepcidin measurements over 48–72 h nor qualified the exact effect of OD or BD dosing on cyclical changes in its concentrations. Elevated basal concentrations of serum hepcidin in patients with CKD lead to a state of functional iron deficiency in CKD in which oral iron absorption and release of stored iron from cellular stores for erythropoiesis are impaired^13^. As hepcidin and ferritin concentrations also increase with inflammation, we have also looked at changes in serum IL-6, a marker of inflammation in CKD. As serum IL-6 concentrations did not change, it is highly unlikely that the change in serum ferritin concentrations in our study were driven by underlying inflammation.

At 12 weeks, MCHC increased in the OD group as compared to decrease in the BD group. It is not surprising that the change in MCHC was not statistically significant when overall study population was considered as the directions of change between the groups were opposite. MCHC is a calculated value and expressed in g/dL as a ratio of hemoglobin to hematocrit. It represents the average concentration of hemoglobin per unit of volume of red blood cell (RBC). MCHC has been traditionally regarded as a reliable marker of availability of iron within the preceding 3–4 months and its incorporation in intracellular hemoglobin^8,14^. A study by Robertson and MaClean in 1970 had suggested that latent iron deficiency should be looked for in otherwise normal, non-anemic women with low MCHC^15^. Over last few decades, variability in MCHC measurements between automated hematology platforms have led to restriction in use of MCHC to just a quality control parameter^16,17^. In fact, this variability in measurement across platforms has been cited as one of the reasons for ‘why MCHC lost the race to be a reliable marker of hypochromia’ in the current era of automated counter measurements^16^. We have measured MCHC on Sysmex XN-1000™ Hematology Analyzer (Sysmex Corporation, Kobe, Japan). The MCHC measurements were done in freshly collected blood samples. In this regard, it is worth noting that it is well documented that MCHC, as measured on automated Sysmex platforms has highest correlation (R^2^: 0.729 versus R^2^ between 0.178 to 0.556 for others) with traditionally calculated ‘true’ MCHC^16–18^. In fact, MCHC measurements done on this platform can be used for clinical assessment and monitoring of anemias just like microhematocrit based ‘true MCHC’ measurements of the past^16^. In the present study, we have done serial measurements on the same platform over 12 weeks. Therefore, difference in MCHC between groups might suggest that incorporation of iron in hemoglobin might be different between groups. However, this would need further exploration is a properly designed study to ascertain whether this is indeed an effect of dosing regimen rather than other factors.

With iron repletion, changes in MCV and hemoglobin lag changes in hemoglobin content within RBCs as reflected by MCH and MCHC^19^. %HYPO-He and RET-He are newer biomarkers of hypochromia that reflect iron deficiency and iron availability for erythropoiesis^8^. RET-He < 29 pg suggests deficient erythropoiesis and Hypo-He > 2.7% has been shown to correlate with iron deficiency in patients on hemodialysis^8,20^. However, there is lack of agreement on universal clinical decision limits for these markers in patients with CKD which has been highlighted in the recent KDIGO controversies conference statement^5^. Different commercially available automated hematology platforms measure these parameters by different techniques and report by different names^8,21,22^. Lack of standardization across platforms, limited availability of these platforms, and no universal agreement on values are major barriers to routine use of these parameters^5,22^. In our study population, the baseline values for these parameters in both the groups were within the normal ranges as reported for general population (Sysmex platform)^8,20^. This is not surprising as the selection criteria for the study population were not based on iron deficient erythropoiesis as defined by these parameters. In fact, study population was selected based on prevalent KDIGO clinical practice recommendations for iron supplementation in CKD^4^. If we look at the trends of estimated marginal means for parameters that reflect hemoglobinization of RBCs, changes in MCH, MCHC and RET-He were more in the OD group as compared to the BD group over 12 weeks (Fig. 2). Though these did not reach statistical significance, these might point towards differences in overall short-term and long-term iron incorporation into developing erythrocytes between the groups. It is worth mentioning that in a review of role of red cell indices in classification and treatment of anemia published in 2013, the authors referred to MCH as a forgotten but clinical useful index^23^. In a recent review published in 2021, MCHC was tabulated as one of the biomarkers of functional iron deficiency^24^. Our study was not designed to detect clinically significant changes in these parameters. As one of the largest trials that investigated oral iron supplementation (ferrous sulphate 100 mg twice daily) in pre-dialysis CKD, FIND-CKD study had shown that intravenous ferric carboxymaltose targeted to higher ferritin concentrations maintained hemoglobin better than oral iron over 56 weeks^25^. Therefore, the impact of different oral iron dosing need to be tested not only over longer duration but also against intravenous regimens so that meaningful clinical conclusions can be drawn.

Our study has several strengths. The study was a randomized, controlled trial. We did serial measurements at baseline, 2, 5 and 12 weeks on the same platform. We selected participants according to the prevalent KDIGO clinical practice guideline, which is more aligned with practice. We have excluded patients who would have required erythropoietin therapy or would have been at risk of poor iron absorption or iron loss from gut. However, lack of a placebo arm, open-label design, restriction to single center and short duration of follow up are few limitations of our study. We have also not measured other biomarkers of iron deficiency like serum soluble transferrin receptor. Also, wide entry criteria with respect to iron status and already higher hemoglobin levels at baseline might have led to limited hemoglobin response in the study population.

In our study, we did not find any significant difference in %TSAT between the groups. Differences in secondary outcomes like ferritin or MCHC between groups are just hypothesis generating. These observations need to explored in patients with more objectively defined true or functional iron deficiency so that effects of less frequent oral iron dosing on long term clinical end points in patients with iron deficiency anemia and CKD can be uncovered.

## Supplementary Information

Supplementary Information 1.

## Data Availability

The datasets analysed during this study are available from the corresponding author on reasonable request.
