# Supplementary material for: A randomized trial of once daily versus twice daily dosing of oral iron in CKD
Source: Sci Rep. 2023 Jan 4;13:141. doi: 10.1038/s41598-022-26589-x (PMC9813132; doi:10.1038/s41598-022-26589-x)
Supplement: Supplementary file 1 — Supplementary Information 1. [file 41598_2022_26589_MOESM1_ESM.docx]

**Supplementary table 1: Change in hematological and biochemical parameters at 2 weeks, 5 weeks and 12 weeks in the OD group**

| **Parameters** | **Baseline** | **2 weeks** | **5 weeks** | **12 weeks** |
| --- | --- | --- | --- | --- |
|  | **Mean** ± **SD /**  **Median (25^th^, 75^th^)** | **Mean** ± **SD /**  **Median (25^th^, 75^th^)** | **Mean** ± **SD /**  **Median (25^th^, 75^th^)** | **Mean** ± **SD /**  **Median (25^th^, 75^th^)** |
| % TSAT | 20.53  (14.00, 25.00) | 20.00  (17.00, 26.75) | 21.00  (14.50, 28.00) | 23.00  (17.00, 32.99) |
| Serum ferritin (µg/L) | 77.85  (36.71, 153.51) | 50.00  (28.50, 92.75) | 66.00  (36.50, 114.00) | 85.00  (50.00, 102.60) |
| Serum iron (µg/dL) | 71.27 ± 28.39 | 83.18 ± 26.79 | 83.92 ± 33.08 | 82.78 ± 30.09 |
| TIBC (µg/dL) | 377  (297, 384) | 380  (375, 387) | 386  (377, 400) | 372  (315, 396) |
| Hemoglobin (g/dL) | 12.59 ± 1.67 | 12.66 ± 1.71 | 12.55 ± 1.66 | 12.61 ± 1.62 |
| MCV (fL) | 87.30  (83.15, 90.22) | 87.30  (83.43, 90.38) | 87.20  (85.60, 88.95) | 87.60  (84.85, 89.55) |
| MCH (pg) | 27.65  (25.73, 28.68) | 27.65  (25.65, 28.75) | 27.90  (26.6, 28.6) | 28.20  (27, 28.75) |
| MCHC (g/dL) | 31.38 ± 1.16 | 31.39 ± 1.22 | 31.61 ± 1.15 | 31.88 ± 1.25 |
| % HYPO-He | 0.31  (0.18, 0.88) | 0.20  (0.13, 0.88) | 0.20  (0.20, 0.55) | 0.30  (0.20, 0.60) |
| RET-He (pg) | 30.65  (29.05, 31.75) | 31.60  (30.08, 32.88) | 32.00  (30.65, 33.05) | 31.70  (30.40, 32.90) |
| Serum IL-6 (pg/mL) | 2.86 (1.27, 7.50) | - | - | 5.74 (1.77, 11.53) |
| Serum hepcidin (ng/mL) | 16.51  (8.44, 23.29) | - | - | 53.51  (38.22, 79.00) |
| Serum creatinine (mg/dL) | 1.79  (1.55, 2.58) | 1.79  (1.43, 2.30) | 1.80  (1.46, 2.30) | 1.90  (1.52, 2.50) |
| UPCR (mg/g) | 0.30  (0.13, 0.95) | 0.36  (0.14, 0.68) | 0.35  (0.16, 0.71) | 0.46  (0.205, 1.00) |
| eGFR (mL/min/1.73m^2^) | 39.51 ± 12.35 | - | - | 38.57 ± 15.73 |

Data presented as mean ± standard deviation or median (25th, 75th percentile) as appropriate. Compared using paired t test.

eGFR: estimated glomerular filtration rate, % HYPO-He: percentage of hypochromic red blood cells, IL-6: interleukin-6, MCH: mean corpuscular hemoglobin, MCHC: mean corpuscular hemoglobin concentration, MCV: mean corpuscular volume, RET-He: reticulocyte hemoglobin equivalent, TIBC: total iron binding capacity, % TSAT: percentage transferrin saturation, UPCR: urine protein/creatinine ratio

**Supplementary table 2: Change in hematological and biochemical parameters at 2 weeks, 5 weeks and 12 weeks in the BD group**

| **Parameters** | **Baseline** | **2 weeks** | **5 weeks** | **12 weeks** |
| --- | --- | --- | --- | --- |
|  | **Mean** ± **SD /**  **Median (25^th^, 75^th^)** | **Mean** ± **SD /**  **Median (25^th^, 75^th^)** | **Mean** ± **SD /**  **Median (25^th^, 75^th^)** | **Mean** ± **SD /**  **Median (25^th^, 75^th^)** |
| % TSAT | 19.45  (15.25, 24.75) | 18.00  (15.00, 28.00) | 23.00  (17.00, 27.00) | 29.00  (22.00, 33.00) |
| Serum ferritin (µg/L) | 67.50  (46.58, 118.25) | 73.00  (37.00, 97.00) | 96.00  (53.00, 170.00) | 103.00  (69.60, 211.00) |
| Serum iron (µg/dL) | 69.44 ± 21.66 | 78.51±32.28 | 84.33± 29.82 | 102.05 ± 31.45 |
| TIBC (µg/dL) | 380  (337, 386) | 385  (367, 390) | 380  (328, 390) | 365  (310, 390) |
| Hemoglobin (g/dL) | 11.91 ± 1.25 | 11.72 ± 1.38 | 11.76 ± 1.49 | 11.93 ± 1.46 |
| MCV (fL) | 86.65  (82.83, 89.68) | 86.80  (82.20, 90.80) | 87.80  (83.80, 91.70) | 88.90  (85.60, 92.50) |
| MCH (pg) | 27.30  (26.65, 28.88) | 27.50  (26.20, 29.10) | 27.50  (26.30, 28.80) | 27.60  (26.60, 29.30) |
| MCHC (g/dL) | 31.75 ± 1.17 | 31.62 ± 1.14 | 31.50 ± 1.16 | 31.25 ± 1.08 |
| % HYPO-He | 0.36  (0.20, 0.90) | 0.31  (0.20, 0.70) | 0.20  (0.10, 0.70) | 0.30  (0.10, 0.60) |
| RET-He (pg) | 30.53  (29.18, 31.68) | 31.10  (29.70, 32.70) | 31.30  (29.90, 33.00) | 31.40  (30.20, 32.70) |
| Serum IL-6 (pg/mL) | 3.33  (1.87, 11.78) | - | - | 5.56  (2.46, 18.44) |
| Serum hepcidin (ng/mL) | 18.52  (8.96, 25.34) | - | - | 61.12  (38.75, 82.10) |
| Serum creatinine (mg/dL) | 2.13  (1.72, 2.48) | 1.80  (1.50, 2.40) | 1.80  (1.40, 2.50) | 2.11  (1.54, 2.83) |
| UPCR (mg/g) | 0.54  (0.17, 1.74) | 0.32  (0.15, 1.96) | 0.32  (0.16, 1.05) | 0.37  (0.24, 1.13) |
| eGFR (mL/min/1.73m^2^) | 33.79 ± 11.09 | - | - | 34.27 ± 16.31 |

Data presented as mean ± standard deviation or median (25th, 75th percentile) as appropriate. Compared using paired t test.

eGFR: estimated glomerular filtration rate, % HYPO-He: percentage of hypochromic red blood cells, IL-6: interleukin-6, MCH: mean corpuscular hemoglobin, MCHC: mean corpuscular hemoglobin concentration, MCV: mean corpuscular volume, RET-He: reticulocyte hemoglobin equivalent, TIBC: total iron binding capacity, % TSAT: % transferrin saturation, UPCR: urine protein/creatinine ratio

**Supplementary table 3: Parameter estimates of the linear mixed effect model for change in measured parameters in the study population**

| **Parameters** | **Coefficients** | **Confidence Interval** | **p-value** |
| --- | --- | --- | --- |
| % TSAT | | | |
| TSAT baseline | 0.29 | 0.07, 0.50 | 0.008 |
| Week 2 ® |  |  |  |
| Week 5 | 0.73 | -1.77, 3.22 | 0.57 |
| Week 12 | 4.33 | 1.84, 6.83 | 0.001 |
| Serum ferritin (µg/L) | | | |
| Serum ferritin baseline | 0.26 | 0.12, 0.39 | <0.001 |
| Week 2 ® |  |  |  |
| Week 5 | 0.37 | 0.23, 0.52 | <0.001 |
| Week 12 | 0.56 | 0.42, 0.71 | <0.001 |
| Serum iron (µg /dL) | | | |
| Serum Iron baseline | 0.32 | 0.12, 0.51 | 0.002 |
| Week 2 ® |  |  |  |
| Week 5 | 3.18 | -4.88, 11.25 | 0.44 |
| Week 12 | 11.72 | 3.65, 19.79 | 0.004 |
| TIBC (µg/dL) | | | |
| TIBC baseline | -0.02 | -0.19, 0.15 | 0.82 |
| Week 2 ® |  |  |  |
| Week 5 | 0.01 | -0.08, 0.10 | 0.83 |
| Week 12 | -0.10 | -0.19, -0.01 | 0.03 |
| Hemoglobin (g/dL) | | | |
| Hemoglobin baseline | 0.89 | 0.76, 1.02 | <0.001 |
| Week 2 ® |  |  |  |
| Week 5 | 0.05 | -0.12, 0.23 | 0.57 |
| Week 12 | 0.17 | -0.01, 0.35 | 0.06 |
| MCV (fL) | | | |
| MCV baseline | 0.73 | 0.65, 0.80 | <0.001 |
| Week 2 ® |  |  |  |
| Week 5 | 0.16 | -0.50, 0.82 | 0.63 |
| Week 12 | 0.85 | 0.19, 1.51 | 0.01 |
| MCH (pg) | | | |
| MCH baseline | 0.68 | 0.59, 0.76 | <0.001 |
| Week 2 ® |  |  |  |
| Week 5 | 0.06 | -0.19, 0.31 | 0.62 |
| Week 12 | 0.28 | 0.04, 0.53 | 0.03 |
| MCHC (g/dL) | | | |
| MCHC baseline | 0.64 | 0.50, 0.77 | <0.001 |
| Week 2 ® |  |  |  |
| Week 5 | 0.04 | -0.20, 0.28 | 0.73 |
| Week 12 | 0.05 | -0.18, 0.29 | 0.66 |
| % HYPO-He | | | |
| % HYPO-He baseline | 0.73 | 0.62, 0.84 | <0.001 |
| Week 2 ® |  |  |  |
| Week 5 | -0.15 | -0.31, 0.01 | 0.08 |
| Week 12 | -0.15 | -0.31, 0.01 | 0.07 |
| RET-He (pg) | | | |
| RET-He baseline | 0.51 | 0.36, 0.66 | <0.001 |
| Week 2 ® |  |  |  |
| Week 5 | 0.28 | -0.05, 0.61 | 0.10 |
| Week 12 | 0.29 | -0.04, 0.62 | 0.08 |
| Serum creatinine (mg/dL) | | | |
| Serum creatinine baseline | 0.93 | 0.78, 1.09 | <0.001 |
| Week 2 ® |  |  |  |
| Week 5 | 0.02 | -0.01, 0.05 | 0.26 |
| Week 12 | 0.10 | 0.07, 0.13 | <0.001 |
| UPCR (mg/g) | | | |
| UPCR baseline | 0.58 | 0.47, 0.69 | <0.001 |
| Week 2 ® |  |  |  |
| Week 5 | -0.08 | -0.28, 0.12 | 0.42 |
| Week 12 | 0.12 | -0.08, 0.32 | 0.23 |

Compared using the linear mixed effect model.

®: reference

% HYPO-He: percentage of hypochromic red blood cells, MCH: mean corpuscular hemoglobin, MCHC: mean corpuscular hemoglobin concentration, MCV: mean corpuscular volume, RET-He: reticulocyte hemoglobin equivalent, TIBC: total iron binding capacity, % TSAT: percentage transferrin saturation, UPCR: urine protein/creatinine ratio

**Supplementary table 4: Adverse events reported in study participants**

| **Event** | **Number of patients who**  **reported these side effects (n)** | | **p-value** |
| --- | --- | --- | --- |
|  | **OD** | **BD** |  |
| Nausea | 09 | 13 | 0.32 |
| Abdominal pain | 06 | 04 | 0.74 |
| Vomiting | 02 | 04 | 0.68 |
| Constipation | 06 | 07 | 0.76 |

Data presented as number. Compared using Chi-square test/Fisher-exact test
